# Directional selection and the evolution of breeding date in birds, revisited: Hard selection and the evolution of plasticity

**DOI:** 10.1002/evl3.279

**Published:** 2022-02-28

**Authors:** Jarrod D. Hadfield, Thomas E. Reed

**Affiliations:** ^1^ Institute of Evolutionary Biology, School of Biological Sciences University of Edinburgh Edinburgh EH9 3JT UK; ^2^ School of Biological, Earth and Environmental Sciences University College Cork, Distillery Fields North Mall Cork T23 N73K Ireland

## Abstract

The mismatch between when individuals breed and when we think they should breed has been a long‐standing problem in evolutionary ecology. Price et al. is a classic theory paper in this field and is mainly cited for its most obvious result: if individuals with high nutritional condition breed early, then the advantage of breeding early may be overestimated when information on nutritional condition is absent. Price at al.'s less obvious result is that individuals, on average, are expected to breed later than the optimum. Here, we provide an explanation of their non‐intuitive result in terms of hard selection, and go on to show that neither of their results are expected to hold if the relationship between breeding date and nutrition is allowed to evolve. By introducing the assumption that the advantage of breeding early is greater for individuals in high nutritional condition, we show that their most cited result can be salvaged. However, individuals, on average, are expected to breed earlier than the optimum, not later. More generally, we also show that the hard selection mechanisms that underpin these results have major implications for the evolution of plasticity: when environmental heterogeneity becomes too great, plasticity is selected against, prohibiting the evolution of generalists.

The seasonal timing of important life history decisions, such as when to breed, hibernate, or migrate, are typically under natural selection toward some optimum set by ecological circumstances (Visser et al., 2010; McNamara et al., 2011). This optimum can vary through time or across space (Phillimore et al., 2010, 2016; Tansey et al., 2017; Gamelon et al., 2018; de Villemereuil et al., 2020), particularly under climate change or other anthropogenic stressors, with possible demographic consequences if populations cannot keep pace evolutionarily (Visser, 2008; Gienapp et al., 2013; Vedder et al., 2013; Simmonds et al., 2020). Breeding time in birds, in particular, has received a lot of attention (Perrins, 1970; Verhulst & Nilsson, 2008), given the relative ease with which data can be collected on egg laying/hatching dates and fitness components such as number of fledglings, recruits, or adult survival (Newton et al., 1989; Clutton‐Brock & Sheldon, 2010).

Following Darwin (1896) and Fisher (1930), Price et al. (1988) developed a quantitative genetic model in which the nutritional condition of a bird negatively affects its breeding date and positively affects fitness. This paper is mainly cited for the fact that an unmeasured trait (nutritional condition) affecting both fitness and the focal trait (breeding date) can generate spurious evidence for selection on the focal trait. This result is largely self‐evident, and the more interesting outcome from Price's (1988) model is that at equilibrium the mean breeding date in the population is later than the optimal breeding date. This important point seems to have been largely overlooked (Table 1), perhaps because the reason why it happens is hard to intuit from the brief explanation given:

“*In adjusting the breeding dates toward the optimum, selection gives more weight to females in high nutritional condition because they are more fecund. Since these individuals breed earlier, the majority of the population is shifted to dates later than the optimum*.”(Price et al., 1988)

**Table 1:** Number of papers published between January 2016 ‐ February 2021 citing Price et al. (1988) for each of five contexts. R1 refers to Price et al.'s (1988) result that birds are predicted to breed later than the optimal breeding date. R2 refers to Price et al.'s (1988) result that spurious directional selection for earlier breeding is expected without knowledge about a bird's nutritional condition. C1 refers to early breeders being of higher quality, C2 refers to directional selection for earlier breeding being common (without stating that the evidence for this selection might be spurious), and C3 refers to various generic ideas (e.g., phenology is important) or miscitations. A full list of the papers together with the relevant sentences can be found in the Supporting Information

	R1	R2	C1	C2	C3
Number of Papers	4	23	6	10	24

Price's (1988) model, and its surprising outcome, can be more easily understood by considering models of phenotypic plasticity. Here, nutritional condition is the environmental variable that varies between individuals, and breeding date is the phenotypic trait that responds to the environmental variable with plasticity slope b (set to –1 in Price et al., 1988). Since the optimum breeding date is not a function of the environmental variable in their model, we have maladaptive plasticity: individuals in low nutrition environments are breeding too late and individuals in high nutrition environments are breeding too early (Figure 1A). When the mean breeding date across all nutritional environments coincides with the optimal breeding date, the response to selection for earlier breeding dates within low nutrition environments will be equal to the response to selection for later breeding dates within high nutrition environments. It is tempting to think that the net response to selection will therefore be zero and the population mean will continue to reside at the optimum (Figure 1B). However, when mean absolute fitness varies across nutritional environments, the net response is the sum of the responses within each nutritional environment weighted by the contribution of each environment to the total population (i.e., the phenotype‐independent intrinsic rate of increase) (Tufto, 2015). Consequently, the net response to selection is weighted toward the response in high nutrition environments (Figure 1C). Since the response to selection in high nutrition environments is positive, this causes mean breeding date to evolve to be later than the optimal breeding date (Figure 1D). Put differently: birds in high nutrition territories generate more offspring, which means that on average individuals are better adapted to high nutrition environments simply because more genes have come from, and have been subject to selection in, high nutrition environments. Similar conclusions have been drawn from source–sink models where evolution favors adaptation to environments with high intrinsic growth rates, reinforcing the disparity between sources and sinks (van Tienderen, 1991; Holt & Gaines, 1992).

**Figure 1:**
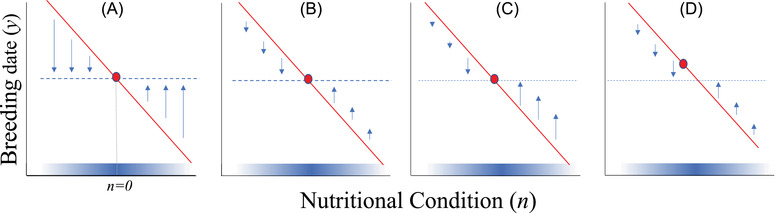
Schematic depiction of the results of Price's (1988) model. A) The strength of selection (indicated by the length of the arrows) increases in more extreme nutritional environments because maladaptive plasticity causes the phenotype (red solid line) to lie further from the optimum (blue dashed line) in these nutritional environments. The mean phenotype (red dot) is assumed to coincide with the optimum when n=0 so the strength of selection is symmetric around n=0. The shaded band of blue in each figure indicates the density of nutritional environments (dark blue ‐ common, light blue ‐ rare). B) Because extreme nutritional environments are rarer, they contribute less to the total response to selection than nutritional environments closer to the mean. However, the contributions to the total response to selection (arrows) are still symmetric around n=0 because the nutritional environment is assumed to be normally distributed with mean zero. C) When α>0 then nutritional environments greater than zero make an increasingly larger contribution to the total response to selection, because they contribute more individuals to the population in the next generation. The contributions to the total response to selection are then not symmetric around n=0 and the positive responses to selection (to the right of n=0) are larger in magnitude than the negative responses to selection (to the left of n=0). D) The intercept of the reaction norm will then evolve to be greater so that the forces of positive and negative selection cancel. The mean phenotype then no longer coincides with the optimum.

The assumption that breeding date depends negatively on nutrition seems untenable when viewed in the light of plasticity models: if the optimum breeding date does not depend on nutrition, then the plasticity slope should evolve to be zero. Under this scenario, the intercept would evolve such that the mean breeding date would fall at the optimum, and nutrition would not generate spurious evidence for selection on breeding date because the two variables would be uncorrelated (Figure 2A). Given there is good evidence that breeding date and nutrition are correlated in birds (Price et al., 1988; Verhulst & Nilsson, 2008), a possible explanation is that the optimal breeding date is a function of nutrition (Verhulst & Nilsson, 2008). Intuition suggests that Price's (1988) central result—that the mean breeding date lies after the mean optimum—is only expected when the plastic slope is more negative than the optimal slope, B (i.e., hyperplasticity; b<B). Under this scenario, the positive selection in high nutrition environments, when weighted by reproductive output, will outweigh the negative selection in low nutrition environments, leading to an increase in the mean breeding date (Figure 2B). However, standard plasticity models, which assume that the phenotype‐independent intrinsic rate of increase does not vary over environments, predict the reaction norm slope should evolve to match the optimal slope b=B (Via & Lande, 1985). The mean breeding date would then be expected to follow the optimum, rather than on average being after it (Figure 2C), although a correlation between nutrition and breeding date would exist if the phenotype‐independent intrinsic rate of increase varies over environments, salvaging the result that Price et al. (1988) is predominantly cited for; i.e., spurious evidence for selection on breeding date. If there is a cost to plasticity (van Tienderen, 1991), or the environments of development and selection are not perfectly correlated (Gavrilets & Scheiner, 1993), then standard plasticity models predict that the plastic slope would lie between 0 and B (“hypoplasticity”). If this result holds when the phenotype‐independent intrinsic rate of increase varies over environments, then following the same logic as above we would expect the mean breeding date to, on average, lie earlier than the optimum (Figure 2D), and spurious evidence for negative directional selection on breeding date to still exist.

**Figure 2:**
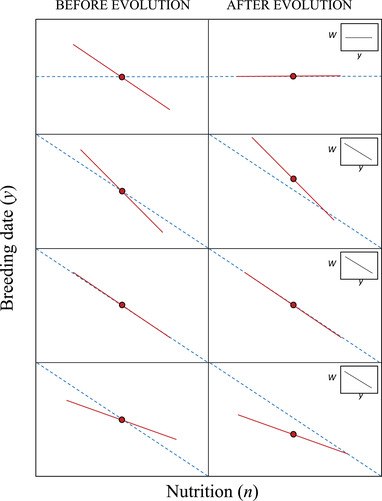
Schematic illustration of different scenarios explained in main text. Blue dashed lines represent optimal breeding time θ, which is independent of individual nutrition n in the top row, and negatively related to n in the other rows (with slope B). Red lines depict linear reaction norms (RN) of breeding time y on n (with intercept i and slope b), with red circles representing mean y. The phenotype‐independent intrinsic rate of increase (mean absolute fitness) is assumed to vary over nutritional environments (α>0). In the left column, different arbitrary starting conditions are assumed, and the right column then shows expected outcomes at evolutionary equilibrium. First (top) row is same as Figure 1 and corresponds to the model of Price et al. (1988) although the slope of the RN is allowed to evolve. Second row: hyperplasticity is assumed, resulting in an upwards shift of the RN (i.e. larger i evolves). Third row: perfect plasticity is assumed (b=B), so no evolution occurs. Fourth row: hypoplasticity is assumed, so intercept of RN shifted downwards (i.e. smaller i evolves). Inset graphs in right column indicate whether a negative correlation between fitness W and breeding time y (i.e. apparent phenotypic selection) exists at equilibrium.

Here, we extend Price's (1988) model to allow the relationship between nutrition and breeding date to evolve, or equivalently we extend plasticity models to cases where the phenotype‐independent intrinsic rate of increase varies over environments. In doing so, we confirm that the results from standard plasticity models outlined in the introduction largely apply in this newcontext.

## Methods

As suggested in the introduction, we will use the notation typical of plasticity models rather than Price at al.'s (1988) original notation (See Table 2).

**Table 2:** Table of symbols used in the model. Parameters may be subscripted to indicate the trait (breeding date y or nutritional condition n) or the reaction norm component (intercept i or slope b)

Symbol	Meaning
y	Breeding date
n	Nutritional condition
i	Breeding date when nutritional condition is zero
b	Plastic response of breeding date to nutritional condition
a	Additive genetic value
e	Environmental value
W	Fitness
α	Effect of nutritional condition on fitness
θ	Optimal breeding date
A	Optimal breeding date when nutritional condition is zero
B	Effect of nutritional condition on optimal breeding date
ω	Strength of stabilising selection (larger values indicate weaker selection)
β	Selection gradient
μ	Mean (in the case of fitness an overbar is used)
$\sigma^{2}$	Variance
σ	Covariance
${}^{*}$	Indicates a quantity at equilibrium

Breeding date y is given by

(1) $$y=a_{i}+\left(a_{b}+e_{b}\right)n+e_{i}$$

where $i=a_{i}+e_{i}$ is the intercept with breeding value $a_{i}$ and environmental value $e_{i}$, and $b=a_{b}+e_{b}$ is the plastic slope for breeding date with respect to nutrition n, with $a_{b}$ and $e_{b}$ being the breeding value and environmental component, respectively. In Price et al. (1988), b is set to −1 for all individuals. All random variables are assumed to be normally distributed and the environmental deviations have zero expectation. Without loss of generality, we also assume that nutritional condition has an expectation of zero.

The optimal breeding date θ depends on n through the function θ=A+Bn and the strength of stabilizing selection around the optimum is inversely proportional to $\omega_{y}^{2}$. In Price et al. (1988), B is assumed to be zero. In addition to the indirect selection acting on plasticity through its effect on the phenotype, we also have direct selection toward a plastic slope of zero with the strength of selection around zero being inversely proportional to $\omega_{b}^{2}$ (van Tienderen, 1991). This represents a maintenance cost to plasticity (Chevin et al., 2010), although we emphasize that other mechanisms that penalize greater plasticity, such as other types of cost, limitations (DeWitt et al., 1998) or imperfect cues (Gavrilets & Scheiner, 1993) are likely to generate the same qualitative conclusions. As in Price et al. (1988), fitness depends log‐linearly on nutrition such that the fitness function is given by

(2) $$W=\exp\left(\alpha n-\frac{{\left(\theta -y\right)}^{2}}{2\omega_{y}^{2}}-\frac{b^{2}}{2\omega_{b}^{2}}\right).$$

When α≠0 the intrinsic rate of increase varies over environments independently of breeding date (the focal phenotype), while the match between breeding date and the optimum for any given n also affects the intrinsic rate of increase. This can be interpreted as hard selection sensu Christiansen (1975), in that each nutritional condition does not contribute equally in demographic terms to the overall population, but rather in proportion to the strength of environment‐specific selection. Christiansen (1975) contrasted this with soft selection, which he defined as occurring when each patch (in his case subpopulation, in our case nutritional condition) contributes in constant proportions to overall population size, because of local density regulation that occurs after selection. Bell et al. (2021) pointed out that these definitions of hard and soft selection are not necessarily the same as Wallace's (1975, 1968), and defined hard selection as occurring when absolute trait value affects absolute fitness, and soft selection as cases where only relative trait value (compared to other conspecifics) matters to absolute fitness. Given that previous plasticity models (Via & Lande, 1985; van Tienderen, 1991; De Jong, 1995; Van Tienderen, 1997; Tufto, 2015) used the terms hard and soft selection in the sense of Christiansen (1975), we here also use them in this context, but acknowledge that our findings in relation to evolution of reaction norm intercept (which are driven by the α parameter) could also occur if n is under soft selection sensu Bell et al. (2021). That is, we make no explicit assumptions about whether absolute or relative nutritional condition affects fitness.

The selection gradients (β) on the intercept and slope within an environment can be found by taking the expectation of Equation 2 with respect to y and b to give mean fitness in environment n ($\bar{W}\left(n\right)$), and then taking the derivative of $ln\bar{W}\left(n\right)$ with respect to the mean intercept and slope respectively (Lande, 1976). Assuming no phenotypic covariance between intercept and slope (see below) this gives:

(3) $$\beta_{\mu_{i}}\left(n\right)=\frac{\left(A+Bn-\mu_{i}-n\mu_{b}\right)\left(\sigma_{b}^{2}+\omega_{b}^{2}\right)+n\mu_{b}\sigma_{b}^{2}}{\omega_{b}^{2}\left(\sigma_{i}^{2}+\omega_{y}^{2}\right)+\sigma_{b}^{2}\left(\sigma_{i}^{2}+n^{2}\omega_{b}^{2}+\omega_{y}^{2}\right)}$$

and

(4) $$\beta_{\mu_{b}}\left(n\right)=\frac{\left(A+Bn-\mu_{i}-n\mu_{b}\right)n\omega_{b}^{2}-\mu_{b}\left(\sigma_{i}^{2}+\omega_{y}^{2}\right)}{\omega_{b}^{2}\left(\sigma_{i}^{2}+\omega_{y}^{2}\right)+\sigma_{b}^{2}\left(\sigma_{i}^{2}+n^{2}\omega_{b}^{2}+\omega_{y}^{2}\right)}$$

where $\sigma_{i}^{2}=\sigma_{a_{i}}^{2}+\sigma_{e_{i}}^{2}$ and $\sigma_{b}^{2}=\sigma_{a_{b}}^{2}+\sigma_{e_{b}}^{2}$. The response to selection in each environment is then given by Lande (1979):

(5) $$\Delta_{\mu_{i}}\left(n\right)=\sigma_{a_{i}}^{2}\beta_{\mu_{i}}\left(n\right)+\sigma_{a_{i},a_{b}}\beta_{\mu_{b}}\left(n\right)$$

(6) $$\Delta_{\mu_{b}}\left(n\right)=\sigma_{a_{b}}^{2}\beta_{\mu_{b}}\left(n\right)+\sigma_{a_{i},a_{b}}\beta_{\mu_{i}}\left(n\right)$$

The total response to selection can be obtained by weighting each environment's response by the frequency of that environment multiplied by relative mean fitness in that environment (relative to the global mean) and then averaging these over environments (Via & Lande, 1985; van Tienderen, 1991; Tufto, 2015):

(7) $$\Delta_{\mu_{i}}=\int \Delta_{\mu_{i}}\left(n\right)\frac{\bar{W}\left(n\right)}{\bar{W}}f\left(n\right)dn$$

(8) $$\Delta_{\mu_{b}}=\int \Delta_{\mu_{b}}\left(n\right)\frac{\bar{W}\left(n\right)}{\bar{W}}f\left(n\right)dn$$

where f(n) is the normal density function with mean zero and variance $\sigma_{n}^{2}$. $\bar{W}$ is the global mean fitness. As long as the absolute genetic correlation between intercept and slope is not one, the equilibrium solutions to Equations 7 and 8 (i.e., when Δ=0) can be found by solving the Equations

(9) $$\beta_{\mu_{i}}=\int \beta_{\mu_{i}}\left(n\right)\frac{\bar{W}\left(n\right)}{\bar{W}}f\left(n\right)dn=0$$

(10) $$\beta_{\mu_{b}}=\int \beta_{\mu_{b}}\left(n\right)\frac{\bar{W}\left(n\right)}{\bar{W}}f\left(n\right)dn=0$$

Equations 9 and 10 cannot be solved analytically. However, Equation 9 can be solved by assuming the phenotypic variance is constant across environments. In reality the phenotypic variance is a quadratic function of n

(11) $$\sigma_{y}^{2}\left(n\right)=\sigma_{i}^{2}+2\sigma_{i,b}n+\sigma_{b}^{2}n^{2}$$

and we approximate this as $\sigma_{y}^{2}\left(n\right)\approx E\left[\sigma_{y}^{2}\left(n\right)\right]=\sigma_{y}^{2}=\sigma_{i}^{2}+\sigma_{b}^{2}\sigma_{n}^{2}$ thereby ignoring the increased strength of selection on the variance in extreme environments. This “constant variance” approximation will be accurate under weak selection and/or when there is little between‐individual variance in the slopes (de Jong, 1999). Solving Equation 10 requires the further approximation that there is little slope variance (i.e., $\sigma_{b}^{2}\approx 0$). The equilibrium solution for the slope is complex and so we also obtain solutions making the further assumption that variation in mean fitness across environments is dominated by α, rather than the mismatch between mean breeding date and the optimum (i.e., $\bar{W}\left(n\right)\propto \exp\left(n\alpha \right)$). This is expected when $|\frac{B-\mu_{b}}{2\omega_{y}^{2}}|$ is small, either because B is small in magnitude, selection on phenotype is weak, or the cost of plasticity is low (since then $B=\mu_{b}$). We refer to this approximation as the “weak mismatch” approximation.

A Mathematica notebook is available on request from the corresponding author.

### SIMULATIONS

To test the validity of the approximations we simulated a population of 10,000 individuals with equal numbers of males and females. Initial breeding values were sampled from a multivariate normal with covariance matrix equal to the genetic (co)variances of the intercept and slope and means equal to the approximate equilibrium solutions. Environmental values and nutritional condition were also sampled from independent normals with zero means and specified variances. Individuals of each sex were sampled with replacement 10,000 times to generate the parental identities of the next generation. The probability of an individual being sampled was proportional to its absolute fitness defined by Equation 2. The breeding values of the new generation were obtained by sampling from a normal with mean equal to the average of their parents' breeding values, and covariance matrix equal to the half the genetic (co)variances, as expected under the infinitesimal model (Barton et al., 2017). The effects of inbreeding were ignored. The population was allowed to evolve for 10,000 generations and the average intercept and slope across the final 5,000 generations was calculated as it was assumed that the population had reached equilibrium by this point. This was done 100 times for B varying between –40 and 0 in 0.4 increments, and with $\sigma_{i}^{2}=\sigma_{b}^{2}=\sigma_{n}^{2}=\alpha =1$, A=0 and $\omega_{y}^{2}=\omega_{b}^{2}=20$. The equilibrium values only depend on the genetic (co)variances through their contribution to the phenotypic (co)variances so we set the heritability of intercepts and slopes to one to speed up the rate of equilibration.

When obtaining expressions for the selection gradients within each environment (β(n)) we assumed that there was no phenotypic covariance between intercept and slope. This is usually justified by the fact that this minimizes the variance in phenotype in the average environment (if the average environment is defined such that it takes value zero) and this canalization is selected for under stabilizing selection (Lande, 2009). However, under the present model, this justification would imply that the phenotypic correlation between intercept and slope should evolve to minimize the phenotypic variance in the average environment weighted by the intrinsic rate of increase. Using simulation, we test the robustness of the approximation to extreme correlations (–0.9 and 0.9) in addition to our assumption of a zero correlation.

Our approximations for the equilibrium slope when the variance in slopes is small do not depend on α, but our weak mismatch approximation suggests that in the presence of slope variance the equilibrium slope will depend on α (see Results). We therefore reran the zero‐correlation simulation detailed above but holding B constant (−1) and allowing α to vary from 0 to 1 in 0.01 increments.

Simulation code can be found in the Supporting Information.

## Results

### ANALYTICAL MODEL

Under the assumption of constant phenotypic variance the equilibrium intercept is

(12) $$\begin{array}{ccc} & & \mu_{i}^{*}=A+\sigma_{n}^{2}\alpha \left(B-\mu_{b}\frac{\omega_{b}^{2}}{\sigma_{b}^{2}+\omega_{b}^{2}}\right)\\ & & \frac{\left(\sigma_{b}^{2}+\omega_{b}^{2}\right)\left(\sigma_{b}^{2}\left(\sigma_{i}^{2}+\omega_{y}^{2}\right)+\omega_{b}^{2}\left(\sigma_{y}^{2}+\omega_{y}^{2}\right)\right)}{\left.\left(\sigma_{b}^{2}+\omega_{b}^{2}\right)\left(\sigma_{b}^{2}(\sigma_{i}^{2}+\omega_{y}^{2}\right)+\omega_{b}^{2}\left(\sigma_{y}^{2}+\omega_{y}^{2}\right)\right)+\sigma_{b}^{2}\omega_{b}^{2}\mu_{b}^{2}\sigma_{n}^{2}}. \end{array}$$

Since the right‐hand quotient must be positive, this demonstrates that the mean breeding date only evolves to be greater than the optimal breeding date when $B>\mu_{b}\frac{\omega_{b}^{2}}{\sigma_{b}^{2}+\omega_{b}^{2}}$. Since $\frac{\omega_{b}^{2}}{\sigma_{b}^{2}+\omega_{b}^{2}}$ cannot be greater than one, this implies hyperplasticity is required when B≤0, and in cases where the strength of selection against plasticity is strong ($\omega_{b}^{2}$ is small compared to $\sigma_{b}^{2}$) the degree of hyperplasticity required may be substantial. It can also be seen that if the plasticity slope is shallower than B, as is expected from most phenotypic plasticity models (see below also), then the mean breeding date will in general lie before the optimum, not after. Assuming there is little slope variance ($\sigma_{b}^{2}\approx 0$), the equilibrium intercept has an even more straightforward form:

(13) $$\mu_{i}^{*}=A+\sigma_{n}^{2}\alpha \left(B-\mu_{b}\right)$$

and any level of hyperplasticity will cause the mean breeding date to be greater than the optimal breeding date. Price et al. (1988) assume B=0 and $\mu_{b}=-1$ hence their result that the mean breeding date exceeds the optimum by an amount $\sigma_{n}^{2}\alpha$ is recovered. Under the assumption of little slope variance, the equilibrium slope is given as

(14) $$\mu_{b}^{*}=\frac{2}{3}B-\frac{1}{3}\frac{pa{\left(\frac{pb+\sqrt{4pa^{3}+pb^{2}}}{2}\right)}^{-1/3}-{\left(\frac{pb+\sqrt{4pa^{3}+pb^{2}}}{2}\right)}^{1/3}}{\sigma_{n}^{2}}$$

where

(15) $$pa=\sigma_{n}^{2}\left(3\left(\sigma_{i}^{2}+\omega_{y}^{2}+\sigma_{n}^{2}\omega_{b}^{2}\right)-\sigma_{n}^{2}B^{2}\right)$$

and

(16) $$pb=-2B\sigma_{n}^{4}\left(9\left(\sigma_{i}^{2}+\omega_{y}^{2}-\sigma_{n}^{2}\omega_{b}^{2}/2\right)+\sigma_{n}^{2}B^{2}\right)$$

**Figure 3:**
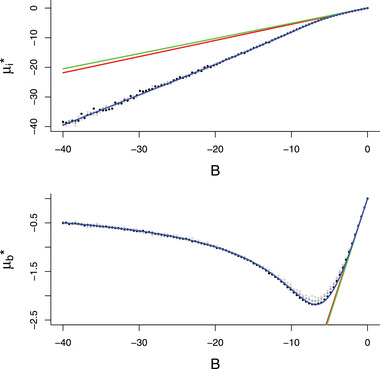
Circles represent the average intercept ($\mu_{i}^{*}$) and slope ($\mu_{b}^{*}$) of 50 million individuals (10,000 from each of the 5,000 final generations of a 10,000 generation simulation). Light grey, dark grey and black circles are for simulations where the correlations between intercepts and slopes are ‐0.9, 0, and 0.9 respectively. The blue line is the approximation assuming the variance in slopes is small ($\sigma_{b}^{2}\approx 0$). The green and red lines assume that variation in mean fitness across nutritional environments is dominated by the direct effect of nutrition on fitness (α), rather than variation in the phenotype‐optimum mismatch (‘weak mismatch'). The green line assumes the phenotypic variance is constant and the red line assumes the variance in slopes is small. Parameter values were set to $\sigma_{i}^{2}=\sigma_{b}^{2}=\sigma_{n}^{2}=\alpha =1$, A=0, and $\omega_{y}^{2}=\omega_{b}^{2}=20$ with the slope of the relationship between the optimal breeding date and nutrition (B) varying.

It can be shown (see the Mathematica notebook for details) that if B=0, as assumed in Price et al. (1988), then the equilibrium slope is zero and any relationship between nutrition and breeding date disappears. In addition, hyperplasticity implies the equilibrium slope must be less than B, if B is negative, but this cannot be satisfied in the above equations. The model therefore demonstrates that hypoplasticity is the expected outcome and the mean breeding date is expected to lie before, not after, the optimal breeding date. In addition to this main result, it should also be noted that the equilibrium slope under this approximation does not depend on α and is not monotonic in B; the slope is maximized at intermediate values of B (see Figure 3 also). In the Supporting Information, we explore the reason for this behavior, and in the discussion give an intuitive explanation as to why it happens.

The solutions given above are complex, but by assuming that variation in mean fitness across environments is dominated by α (“weak mismatch”) simpler answers can be achieved. Under weak mismatch and constant variance, the equilibrium intercept is

(17) $$\mu_{i}^{*}=A+\sigma_{n}^{2}\alpha \left(B-\mu_{b}\frac{\omega_{b}^{2}}{\sigma_{b}^{2}+\omega_{b}^{2}}\right)$$

and the equilibrium slope is

(18) $$\mu_{b}^{*}=B\frac{\sigma_{n}^{2}\omega_{b}^{2}}{\sigma_{n}^{2}\omega_{b}^{2}\left(1+\frac{\sigma_{b}^{2}\sigma_{n}^{2}\alpha^{2}}{\sigma_{b}^{2}+\omega_{b}^{2}}\right)+\sigma_{i}^{2}+\omega_{y}^{2}}$$

Under weak mismatch and little slope variance, the equilibrium intercept

(19) $$\mu_{i}^{*}=A+\sigma_{n}^{2}\alpha \left(B-\mu_{b}\right)$$

is identical to that under the assumption of little slope variance alone (Eq. 13), but the equilibrium slope is

(20) $$\mu_{b}^{*}=B\frac{\sigma_{n}^{2}\omega_{b}^{2}}{\sigma_{n}^{2}\omega_{b}^{2}+\sigma_{i}^{2}+\omega_{y}^{2}}$$

In both cases, hyperplasticity is required for the mean breeding date to be later than the optimum, yet hypoplasticity (or perfect plasticity if selection against plasticity is weak) is expected to evolve. Consequently, all approximations confirm the previous qualitative conclusions: hypoplastcity is expected to evolve and the mean breeding date is displaced to earlier dates than the optimum breeding date. However, note that unlike the approximations where the slope variance is small, the equilibrium slope depends on α under the constant variance approximation with weak mismatch (Eq. 18). This suggests that the equilibrium slope is sensitive to α when there is slope variation (see simulation results). The equilibrium slope under the weak mismatch approximation with little slope variance (Eq. 20) is identical to that found by Lande (2014) (see Supporting Information) since it ignores the consequences of hard selection induced by greater phenotype‐optimum mismatch in extreme environments and is therefore equivalent to a model assuming soft selection on breeding date. Finally, in the approximations where the slope variance is not assumed small, the equilibrium intercept depends on the cost of plasticity even after conditioning on the mean plastic slope (Equations 12 and 17). It is not clear to us why this is the case.

### SIMULATIONS

Using simulations, we confirm that the approximation when the slope variance is small is very accurate under biologically reasonable parameter values, and that the weak mismatch approximations perform well when B is small in magnitude (Figure 3). Phenotypic correlations between intercept and slope appear to have little influence on the equilibrium values, suggesting that results obtained under the assumption that the correlation is zero are likely to be robust. While the equilibrium slope value depends on α when there is slope variation, simulations show that this dependency is very weak (see Supporting Information; a 1% change in equilibrium value for a shift from α=0 to α=1) and the approximation with little slope variance, which does not involve α, remains accurate.

## Discussion

In Price at al.'s (1988) classic model, a negative relationship between a bird's nutritional condition and breeding date is imposed. Since the optimal breeding date is assumed constant across nutritional states, this leads to maladaptive plasticity and begs the question why birds in high nutritional condition would choose to breed earlier than what was optimal? Here, we allowed the relationship between nutritional condition and breeding date to evolve under these conditions (B=0) and showed that the relationship would disappear, taking Price at al.'s (1988) main results with it. However, by assuming the optimal breeding date does in fact become earlier with increasing nutritional condition (B<0), we show that the main result that Price et al. (1988) is cited for can be salvaged: a correlational analysis of fitness and breeding date would incorrectly generate evidence for negative directional selection, if nutritional condition is not controlled for by including it in the analysis (e.g., because it is unmeasured). However, the more interesting and less obvious result in Price et al. (1988) is that the mean breeding date in the population should be later than the optimum breeding date. Here, we show that the exact opposite result is more likely: the mean breeding date in the population should be earlier than the optimum breeding date. This occurs because the plastic response of breeding date to nutrition is likely to be shallower than the optimal response because of a cost to plasticity (or an imperfect cue). Therefore, from the plastic response alone, birds in high nutritional condition breed later than what is optimal to the same degree that birds in low nutritional condition breed earlier than what is optimal. However, because birds with high nutritional condition have intrinsically higher reproductive output, the intercept evolves so that birds in high nutritional condition are better adapted than those in low nutritional condition. This shifts the intercept to earlier values as this reduces the discrepancy between the plastic and optimal responses in high nutritional conditions.

In order to recover Price at al.'s (1988) main result that birds on average breed later than what is optimal, a hyperplastic response to nutrition would be required. While hyperplasticity is not an outcome in our rather simple model, it has been shown to be an outcome under three different scenarios. First, large fluctuations in the optimum that are not predicted by nutritional condition could select for hyperplasticity. This is because the fitness surface may move so much that the population finds itself in a convex region most of the time, where disruptive selection operates. The increased variance that hyperplasticity affords is then selected for, as a result of this disruptive selection (Tufto's (2015) interpretation of Scheiner's (2013) simulations). In birds, there are clearly large annual and spatial shifts in the optimum breeding date that are independent of within‐population variation in nutritional condition of parents; e.g., variation in the timing of peak availability of food fed to chicks (Visser et al., 1998). However, these shifts in the optimum seem to be reasonably well tracked by plasticity (de Villemereuil et al., 2020) (probably in response to temperature (Visser et al., 2006; Charmantier et al., 2008; Phillimore et al., 2016)) and so it is unclear whether the average distance between the optimum and mean breeding date is sufficiently large to select for increased variance and therefore hyperplasticity. Moreover, in such cases a separate variance‐inducing mechanism (diversified bet‐hedging) is likely to evolve, allowing nutrition‐mediated plasticity to evolve to its hypoplastic optimum (Tufto, 2015).

Second, if the optimum breeding date is determined by nutrition, but the plastic response is determined by a correlated cue (the environment of development), then hyperplasticity can evolve when the cue accuracy varies across spatiotemporal scales (King & Hadfield, 2019). The optimal plastic slope at each scale is shallower the lower the cue accuracy at that scale (Gavrilets & Scheiner, 1993), and the evolved slope is a compromise between these scale‐specific optimal slopes. This can generate hyperplasticty at scales where the evolved slope is steeper than the scale‐specific optimal slope. Since the scale of most studies is individuals in a population over short time periods, the between individual relationship between nutrition and the environment of development would have to be weaker than at other scales (such as between‐population or between‐year) for hyperplasticity to be seen. However, the evolved slope is weighted toward those scales at which it is difficult to genetically track shifts in the optimum: scales where spatial fluctuations in the optimum with respect to dispersal distance are short and/or temporal fluctuations in the optimum with respect to generation time are fast. This implies that the evolved slope is going to be especially weighted toward the optimal slope from the short/fast fluctuations present within populations/years and so the discrepancy between the evolved slope and the optimal slope at this scale may not be large enough to generate much hyperplasticity, even if the cue accuracy at this scale was especially weak. Third, hyperplasticity can be selected for when there is assortative mating for a trait for which plastic induction occurs prior to migration and mating and there is sex‐biased dispersal (Ronce pers. comm.). Breeding date, by its very nature, is subject to strong assortative mating, and the requirement for spatial variation in, and local adaptation to, the nutritional environment seems plausible in bird populations. However, the assumption that birds would make plastic decisions about when to breed based on the nutritional environment where they were raised, rather than where they are currently breeding, seems less tenable. Finally, hyperplastic responses could be the result of maladaptation, but this is generally thought to arise when species experience novel environments and so is unlikely to be a widespread phenomenon (Ghalambor et al., 2007).

Given that the known mechanisms for generating hyperplasticity seem rare or unlikely, the hypoplasticity that is likely to evolve leads to our prediction that birds, on average, are breeding earlier than the optimum. Assessing this empirically is difficult because it would require the direct effect of nutritional condition on fitness (determined by α) to be controlled for when estimating the effect of breeding date on fitness. Without doing this, the optimal breeding date is likely to be inferred to be earlier than the observed mean breeding date, suggesting, incorrectly, that birds are breeding later than the optimum. Indeed, it is this very effect that Price et al. (1988) is usually cited for. Moreover, other mechanisms can generate the scenario where the mean phenotype deviates from the mode of the individual fitness surface, such as skewed breeding values (Turelli & Barton, 1994; Rice, 2002; Bonamour et al., 2017) and asymmetric fitness functions (Lof et al., 2012; Urban et al., 2013).

The alternative approach for testing whether the mean breeding date is earlier or later than the optimum is to perform an experiment. This is most often done using replacement clutch experiments or cross‐fostering experiments (Verhulst & Nilsson, 2008). For example, in cross‐fostering experiments, birds that naturally breed on the same date (and therefore have the same expected nutritional condition) are taken and forced to raise chicks at a range of hatch dates by giving them eggs that have been incubated more (so their chicks hatch earlier) or less (so their chicks hatch later). When looking at the fitnesses of birds with the same natural lay date, the evidence suggests that the manipulated hatch date that maximises fitness is less than the mean hatch date, consistent with Price's (1988) model rather than ours (Verhulst & Nilsson, 2008). However, the experimental reduction in breeding time in these experiments is completely confounded with an experimental reduction in the cost of incubation that would produce spurious evidence that the optimum is earlier than the mean breeding date (Verhulst & Nilsson, 2008). Moreover, because hatching date is manipulated rather than breeding date these experiments may miss any fitness costs associated with laying early, such as smaller clutch sizes or increased adult mortality. Since the mean breeding date that evolves maximizes mean fitness, rather than maximizes a component of fitness such as breeding success (Singer & Parmesan, 2010; Johansson & Jonzén, 2012; Visser et al., 2012; Day & Kokko, 2015) this may generate spurious evidence that the mean phenotype does not coincide with optimum. Cleaner manipulations that focus on breeding date rather than hatching date would provide better evidence but are hard to achieve.

Given the issues raised above, it may be easier to test the assumption that our prediction is based on: that any fitness advantage of breeding early is greater for birds in high nutrition. To our knowledge, no study has sought to test this idea directly, although several possible mechanisms exist. For example, if breeding early entails the risk that conditions are too cold to sustain incubation, but high nutrition birds are more confident they can sustain incubation, they may choose to go early so that their chicks hatch early. In contrast, low nutrition birds that are not confident they can sustain incubation may choose to go late even if their chicks hatch later. This relates to the individual optimisation hypothesis (Pettifor et al., 1988, 2001), which is usually applied in the context of clutch size optimization but also relates to date effects (Daan et al., 1988, 1990). Existing data from hatch‐date manipulations could be used to test whether the manipulated hatch date that maximizes fitness depends on original lay date. However, a positive relationship between the inferred optimum date and the actual lay date would merely support individual optimisation of lay date without implicating nutritional condition as the driver. To test this, hatch‐date manipulation experiments could be extended by splitting birds that naturally breed on the same date into two groups and giving one group food supplements and the other not. Birds in both groups could then be subject to hatching date manipulations, and the optimum breeding date estimated. If the key assumption of our model holds (B<0), we expect the inferred optimum breeding date to be earlier in the group that have been food supplemented. This experimental design would, however, miss any changes in the optimum due to changes in pre‐hatching fitness components driven by nutritional condition (for example, birds in high nutrition can lay early with out suffering an increased risk of mortality). In addition, if the cost of incubation is reduced for food‐supplemented birds this will reduce the bias inherent in cross‐fostering experiments for this group, causing what would appear to be a shift in the optimum toward later dates in food supplemented birds, opposite to what we predict.

While we have disagreed with Price et al. (1988) in regard to the likely direction in which breeding dates may deviate from their optima, we do not wish to diminish the importance of Price's (1988) paper in highlighting that the mean phenotype may not coincide with the peak of the individual fitness surface even if it coincides with the peak of the “fitness landscape,” i.e., a plot of mean fitness versus mean phenotype (Fear & Price, 1998; Walsh & Lynch, 2018). This has important implications as breeding‐date manipulations, which aim to estimate parameters of the fitness surface, do not necessarily imply directional selection when the mean breeding date is shown to deviate from the optimum. Similarly, methods such as Lande‐Arnold (1983) regression that aim to estimate parameters of the fitness landscape do not imply that the mean and optimum phenotypes match when no directional selection is found. These points are most evident when converting the parameters of the Gaussian fitness surface into selection gradients (Lande, 1980, Eq. 8): there may be no directional selection on a trait (breeding date) that deviates from it's optimum if there are other traits (nutritional condition) that are also not at their optima and that are correlated and/or under correlational selection with the focal trait.

While the primary focus of this work was to clarify and extend the work of Price et al. (1988), our results also provide new insights into the evolution of plasticity more generally. Specifically, we find that as B gets steeper, mean plasticity increases to allow better tracking of the optimum, as expected. However, if B becomes too steep then we find that plasticity is actually selected against and mean plasticity starts to decrease (see Figure 3). Since our approximation for the equilibrium slope value does not depend on α, and therefore variation in the phenotype‐independent intrinsic rate of increase, it is surprising that this behavior has not been noted before in previous models. The reason for this is that this behavior depends on there being hard selection on the focal trait (i.e., deviations of breeding date from a nutrition‐dependent optimum reduce mean absolute fitness) in the presence of a cost to plasticity. In contrast, previous models have either assumed soft selection in the presence of a cost (e.g., King & Hadfield, 2019), hard selection in the absence of a cost (e.g., Via & Lande, 1985; Tufto, 2015) or constant environmental values among individuals within a generation in the presence of a cost (e.g., Chevin et al., 2010; Lande, 2014; Scheiner et al., 2020). In our model, the reason that plasticity gets weaker as B gets too steep is that mean fitness in extreme environments becomes so low, owing to phenotype‐optimum mismatch, that extreme environments contribute very little to the response to selection and so the population mainly adapts to environments closer to the average. The selection to increase the slope (via phenotype change) in these average environments is weaker than in extreme environments (Lande, 2009) but the selection to decrease the slope (via the cost of plasticity) is the same, and so plasticity decreases. This outcome can be interpreted as the evolution of a specialist (to average environments) in the presence of too much environmental heterogeneity. This result is opposite to that found by Lande (2014) but is found in van Tienderen's (1991) two‐patch model and Holt and Gaines’ (1992) non‐plasticity model, both of which allow for hard selection sensu Christiansen (1975).

In conclusion, we believe the widespread observation that individuals of high nutritional condition breed earlier can most readily be explained by the optimal breeding date being earlier for such individuals (Verhulst & Nilsson, 2008). Without this, plasticity models predict any relationship between nutritional condition and breeding date would disappear. However, when there are costs to plasticity, the plastic response to nutritional condition is shallower than the optimal response, and this leads to individuals, on average, breeding earlier than the optimum when nutritional condition positively influences fitness.

## 1 AUTHOR CONTRIBUTIONS

J.D.H. and T.E.R. conceived of the ideas presented. J.D.H. conducted the analytical work. J.D.H. and T.E.R. wrote the paper.
